# Artificial Intelligence for Smart Manufacturing: Methods and Applications

**DOI:** 10.3390/s21165584

**Published:** 2021-08-19

**Authors:** Kim Phuc Tran

**Affiliations:** GEMTEX Textile Research Laboratory, ENSAIT, University of Lille, 59000 Lille, France; kim-phuc.tran@ensait.fr; Tel.: +33-0320258960

The term Industry 4.0 has become increasingly pervasive in the context of industrial manufacturing and it has been considered the fourth industrial revolution (Henning [1]). The industrial transformation in the fourth industrial revolution is fueling the shift to Smart Manufacturing. By integrating advanced technologies like Industrial Internet of Things (IIoT), Big Data, Cloud Computing, Artificial Intelligence (AI), manufacturing will become intelligent and independently perform complex tasks such as predictive maintenance of machines, monitoring and optimizing the quality of products, see [2,3,4,5] for example. According to [6], the application of the IIoT in Smart Manufacturing could lead to a decrease in: production costs by 10–30%, logistics costs by 10–30% and quality management costs by 10–30%. It is now at the center of Industry 4.0 and attracts a lot of interest from governments, enterprises, and researchers for implementing Smart Manufacturing, see [7] for more details. Recently, an extensive review of technologies for smart manufacturing systems has been conducted in the research of Alcácer and Cruz-Machado [8].

A vital characteristic of the IIoT is that sensors are embedded in all the components related to the manufacturing process. These sensors act as the “senses” for collecting data from the supply, production, storage, distribution, and consumption of products for development in industrial supply chain analysis and optimization, product quality control, and active maintenance [9,10]. Thanks to the data from these processes, advanced computing technologies now perform efficiently and bring intelligence to manufacturing with AI technology. It brings countless advantages to Smart Manufacturing, involving optimization of all stages of the manufacturing process, reducing waste, and creating new smart products and services with high quality. AI technology now plays the role of a “brain” for Smart Manufacturing.

Towards Smart Manufacturing is a long-term and not straightforward process. It requires deep insight into a multiplicity of advanced and modern technologies that are integrated into this process. This Special Issue aims to offer a systematic overview of this research field and provide innovative developments with respect to the current challenges and opportunities for the applications of artificial intelligence in smart manufacturing. It provides a leading forum for disseminating the latest results of theoretical research, technological development, and applications of AI in Smart Manufacturing.

The aim of this Special Issue is to highlight innovative developments with respect to the current challenges and opportunities for the applications of artificial intelligence in smart manufacturing. Topics include but are not limited to the following: real-time monitoring with machine learning and deep learning; artificial intelligence for predictive maintenance; artificial intelligence for smarter cybersecurity; production scheduling with reinforcement learning; artificial intelligence and robotics in smart manufacturing; IoT-enabled smart manufacturing; digital twin-driven smart manufacturing.

This Special Issue uncovers fundamental principles and recent developments in the applications of artificial intelligence in smart manufacturing. The Special Issue contains 19 papers. It attempts to cover the issues related to key enabling technologies for smart manufacturing such as product quality inspection based on deep learning, remaining useful life prediction for predictive maintenance based on deep learning, Machine Vision Systems, intelligent recommender system, Intelligent Decision-Making of Scheduling for Dynamic Permutation Flowshop via Deep Reinforcement Learning, Real-Time and Explainable Process Monitoring, Intelligence-Driven Decision Support System. These contributions represent an advance in the state-of-the-art of key enabling technologies for smart manufacturing [11,12,13,14,15,16,17,18,19,20,21,22,23,24,25,26,27,28,29]. The richness and diverseness of the papers submitted to this Special Issue confirm the importance of applications of AI in Smart Manufacturing. The hope is that the research ideas, results and achievements will inspire active researchers in this field and will contribute to the further development of this important domain.

## Data Availability

Not applicable.
